# ﻿Morphological and molecular identification for four new wood-inhabiting species of *Lyomyces* (Basidiomycota) from China

**DOI:** 10.3897/mycokeys.110.133108

**Published:** 2024-10-30

**Authors:** Qi Yuan, Yunchao Li, Yunfei Dai, Kunyan Wang, Yixuan Wang, Changlin Zhao

**Affiliations:** 1 The Key Laboratory of Forest Resources Conservation and Utilization in the Southwest Mountains of China Ministry of Education, Key Laboratory of National Forestry and Grassland Administration on Biodiversity Conservation in Southwest China, Yunnan Provincial Key Laboratory for Conservation and Utilization of In-forest Resource, Southwest Forestry University, Kunming 650224, China; 2 College of Forestry, Southwest Forestry University, Kunming 650224, China; 3 Yunnan Yunzhihuang Health Technology Co., LTD, Kunming 650224, China

**Keywords:** Biodiversity, phylogenetic analyses, taxonomy, Yunnan Province

## Abstract

Fungi are one of the most diverse groups of organisms on Earth, in which the wood-inhabiting fungi play an important role in forest ecosystem processes and functions. Four new wood-inhabiting fungi, *Lyomyceshengduanensis*, *L.niveomarginatus*, *L.wumengshanensis* and *L.zhaotongensis*, are proposed, based on morphological features and molecular evidence. *Lyomyceshengduanensis* differs in the brittle basidiomata with pruinose hymenial surface, a monomitic hyphal system and ellipsoid basidiospores (3.5–6 × 3–4.5 µm). *Lyomycesniveomarginatus* is distinguished by the subceraceous basidiomata with crackled hymenial surface, a monomitic hyphal system and ellipsoid basidiospores (4.5–7 × 3–4 µm). *Lyomyceswumengshanensis* is distinguished by the grandinioid hymenial surface, a monomitic hyphal system and ellipsoid to broad ellipsoid basidiospores (4–6 × 3–5 µm). *Lyomyceszhaotongensis* is unique in the grandinioid hymenial surface, a monomitic hyphal system and broadly ellipsoid basidiospores measuring as 2.6–3.5 × 2.5–3 µm. Sequences of ITS and nLSU rRNA markers of the studied samples were generated and phylogenetic analyses were performed using the Maximum Likelihood, Maximum Parsimony and Bayesian Inference methods. The phylogram, based on the ITS+nLSU rDNA gene regions, included three genera within the Schizoporaceae viz. *Fasciodontia*, *Lyomyces* and *Xylodon*, in which the four new species were grouped into *Lyomyces*. The phylogenetic tree inferred from the ITS sequences highlighted that *L.hengduanensis* group with *L.zhaotongensis* and then closely grouped with *L.crustosus*, *L.ochraceoalbus*, and *L.vietnamensis*. The new taxon *L.niveomarginatus* was retrieved as a sister to *L.juniperi*. The new species *L.wumengshanensis* was sister to *L.macrosporus*. The new taxon *L.zhaotongensis* grouped with *L.hengduanensis* and then closely grouped with *L.crustosus*, *L.ochraceoalbus* and *L.vietnamensis*.

## ﻿Introduction

Fungi are one of the most diverse groups of organisms on Earth and play an indispensable role in the forest ecosystem processes and functioning (Hyde 2022; Guan et al. 2023; Deng et al. 2024a). The wood-inhabiting fungal family Schizoporaceae Jülich includes many variations of the fruiting body types within the order Hymenochaetales Oberw. (Larsson et al. 2006; Wu et al. 2022a; Guan et al. 2023; Zhang et al. 2024) and it comprises a number of representative wood-inhabiting fungal taxa, including diverse hymenophoral morphologies as hydnoid, corticioid and polyporoid (Yurchenko and Wu 2016; Riebesehl and Langer 2017; Yurchenko et al. 2017; Cui et al. 2019; Riebesehl et al. 2019; Jiang et al. 2021; Wu et al. 2022a, 2022b; Guan et al. 2023; Deng et al. 2024a, b; Zhang et al. 2024). In addition, taxa of the family Schizoporaceae are widely found in different continents, causing white rot (Langer 1994; Luo et al. 2022; Guan et al. 2023; Zhang et al. 2024).

The genus *Lyomyces* P. Karst. is typified by *L.sambuci* (Pers.) P. Karst. It is characterised by the resupinate-to-effused basidiomata with a smooth-to-odontioid hymenophore, a monomitic hyphal system with generative hyphae bearing clamp connections, the presence of several types of cystidia and with smooth, thin- to slightly thick-walled basidiospores (Karsten 1881; Bernicchia and Gorjón 2010). Based on the MycoBank database (http://www.mycobank.org, accessed on 25 April 2024) and the Index Fungorum (http://www.indexfungorum.org, accessed on 25 April 2024), *Lyomyces* has 55 specific and infraspecific names registered, of which approximately 41 species of *Lyomyces* are currently known (Rabenhorst 1851; Karsten 1881; Karsten 1882; Cunningham 1959; Cunningham 1963; Wu 1990; Hjortstam and Ryvarden 2009; Xiong et al. 2009; Dai 2010; Dai 2011; Yurchenko and Wu 2013; Gafforov et al. 2017; Riebesehl and Langer 2017; Yurchenko et al. 2017; Chen and Zhao 2020; Yurchenko et al. 2020; Luo et al. 2021b; Luo et al. 2021c; Viner et al. 2022; Guan et al. 2023).

On the basis of the frequent inclusion of data from DNA sequences in many phylogenetic studies, the classification of the wood-inhabiting fungi has been updated continuously (Yurchenko et al. 2020). These pioneering research studies into the family Schizoporaceae were just the prelude to the molecular systematics period (Guan et al. 2023; Zhang et al. 2024). The genus *Hyphodontia* s.l. was indicated to be a polyphyletic group, in which the genera *Xylodon* (Pers.) Gray and *Kneiffiella* P. Karst. included the largest number of species (Yurchenko and Wu 2016; Riebesehl and Langer 2017; Riebesehl et al. 2019). Due to the lack of sequences of some wood-inhabiting fungal taxa, it is difficult to clearly distinguish many genera in this family Schizoporaceae using molecular data; therefore, a broad concept of *Hyphodontia* s.l. was accepted (Yurchenko and Wu 2016; Riebesehl and Langer 2017; Wang and Chen 2017; Riebesehl et al. 2019). Based on the nuclear DNA sequence data, six well-distinguished clades as *Hastodontia* clade, *Hyphodontia* clade, *Lagarobasidium* clade, *Kneiffiella*-*Alutaceodontia* clade, *Xylodon*-*Lyomyces*-*Rogersella* clade and *Xylodon*-*Schizopora*-*Palifer* clade, were included, based on the phylogenetical studies for *Hyphodontia* s.l., in which the genus *Lyomyces* was nested within the *Xylodon*-*Lyomyces*-*Rogersella* clade (Yurchenko and Wu 2013). The research revealed that *Hyphodontia* s.l. was divided into six genera, viz., *Hastodontia* (Parmasto) Hjortstam & Ryvarden, *Hyphodontia* J. Erikss., *Kneiffiella*, *Lagarobasidium* Jülich, *Lyomyces* and *Xylodon*, in which 35 new combinations were proposed, including fourteen *Lyomyces* species (Riebesehl and Langer 2017). On the basis of the sequences of the internal transcribed spacer (ITS) and the nuclear large subunit (nLSU) ribosomal DNA gene, the phylogenetic analysis clarified that the *Lyomycessambuci* complex divided into four new species (Yurchenko et al. 2017). Riebesehl et al. (2019) clarified the generic concept and their phylogenetic reconstruction of *Lyomyces* and the species *L.sambuci* was sister to *L.crustosus* (Pers.) P. Karst (Riebesehl et al. 2019). Based on a combination of the morphological and molecular evidence, the fungal diversity of the family Schizoporaceae was analysed, in which six new species were described: *L.fissuratus* C.L. Zhao, *L.fumosus* C.L. Zhao, *L.niveus* C.L. Zhao, *L.ochraceoalbus* C.L. Zhao, *L.albopulverulentus* C.L. Zhao and *L.yunnanensis* (Luo et al. 2021b, 2021c; Guan et al. 2023).

During the investigations of the wood-inhabiting fungi, we collected four new Hymenochaetales taxa from Yunnan Province, China, that could not be assigned to any described species of the order. We present the morphological characteristics and phylogenetic analyses with ITS and nLSU that support the four species in the genus *Lyomyces*.

## ﻿Materials and methods

### ﻿Morphology

Fresh basidiomata of the fungi growing on the angiosperm branch were collected from the Honghe, Lincang, Puer, Wenshan and Zhaotong of Yunnan Province, P.R. China after recording important information (Rathnayaka et al. 2024). Specimens were dried in an electric food dehydrator at 40 °C (Hu et al. 2022), then sealed and stored in an envelope bag and deposited in the Herbarium of the Southwest Forestry University (SWFC), Kunming, Yunnan Province, P.R. China. Macromorphological descriptions were based on field notes and photos were captured in the field and lab. Colour terminology follows Petersen (Petersen 1996). Micromorphological data were obtained from the dried specimens when observed under a light microscope following the previous study (Guan et al. 2023). The following abbreviations are used: KOH = 5% potassium hydroxide water solution, CB = Cotton Blue, CB– = acyanophilous, IKI = Melzer’s Reagent, IKI– = both inamyloid and indextrinoid, L = mean spore length (arithmetic average for all spores), W = mean spore width (arithmetic average for all spores), Q = variation in the L/W ratios between the specimens studied and n = a/b (number of spores (a) measured from given number (b) of specimens).

### ﻿Molecular phylogeny

The EZNA HP Fungal DNA Kit (Omega Biotechnologies Co., Ltd., Kunming, China) was used to extract DNA with some modifications from the dried specimens. The nuclear ribosomal ITS region was amplified with primers ITS5 and ITS4 (White et al. 1990). The PCR procedure for ITS was as follows: initial denaturation at 95 °C for 3 min, followed by 35 cycles at 94 °C for 40 s, 58 °C for 45 s and 72 °C for 1 min and a final extension of 72 °C for 10 min. The nuclear nLSU region was amplified with primer pair LR0R and LR7 (Rehner and Samuels 1994). The PCR procedure for nLSU was as follows: initial denaturation at 94 °C for 1 min, followed by 35 cycles at 94 °C for 30 s, 48 °C for 1 min and 72 °C for 1.5 min and a final extension of 72 °C for 10 min. The PCR procedure for ITS and nLSU followed the previous study (Zhao and Wu 2017). All newly-generated sequences were deposited in NCBI GenBank (https://www.ncbi.nlm.nih.gov/genbank/) (Table 1).

**Table 1.** List of species, specimens, and GenBank accession numbers of sequences used in this study.

Species name	Specimen No.	GenBank accession No.		References
		ITS	nLSU	
*Fasciodontiabrasiliensis*	MSK-F 7245a	MK575201	MK598734	Yurchenko et al. (2020)
*F.bugellensis*	KAS-FD 10705a	MK575203	MK598735	Yurchenko et al. (2020)
*F.bugellensis*	MSK-F 7353	MK575205	MK598736	Yurchenko et al. (2020)
*F.yunnanensis*	CLZhao 6280	MK811275	MZ146327	Luo and Zhao (2021)
*F.yunnanensis*	CLZhao 6385	MK811277	—	Luo and Zhao (2021)
*Hymenochaeteochromarginata*	He 47	KU978861	JQ279666	Unpublished
*H.rubiginosa*	He 458	JQ279580	—	He and Li (2013)
*Lyomycesalbopulverulentus*	CLZhao 21478	OP730712	OP730724	Guan et al. (2023)
*L.allantosporus*	KAS-GEL4933	KY800401	—	Yurchenko et al. (2017)
*L.allantosporus*	FR-0249548	KY800397	—	Yurchenko et al. (2017)
*L.bambusinus*	CLZhao 4831	MN945968	—	Chen and Zhao (2020)
*L.bambusinus*	CLZhao 4808	MN945970	—	Chen and Zhao (2020)
*L.cremeus*	CLZhao 4138	MN945974	—	Chen and Zhao (2020)
*L.cremeus*	CLZhao 8295	MN945972	—	Chen and Zhao (2020)
*L.crustosus*	TASM:YG G39	MF382993	—	Gafforov et al. (2017)
*L.crustosus*	UC2022841	KP814310	—	Unpublished
*L.densiusculus*	Ryvarden 44818	OK273853	—	Viner et al. (2022)
*L.elaeidicola*	LWZ20180411-20	MT319458	—	Wang et al. (2021)
*L.elaeidicola*	LWZ20180411-19	MT319457	—	Wang et al. (2021)
*L.erastii*	TASM:YG 022	MF382992	—	Gafforov et al. (2017)
*L.erastii*	23cSAMHYP	JX857800	—	Unpublished
*L.fimbriatus*	Wu910620-7	MK575209	—	Yurchenko et al. (2020)
*L.fimbriatus*	Wu911204-4	MK575210	—	Yurchenko et al. (2020)
*L.fissuratus*	CLZhao 4352	MW713742	—	Luo et al. (2021b)
*L.fissuratus*	CLZhao 4291	MW713738	—	Luo et al. (2021b)
*L.fumosus*	CLZhao 8188	MW713744	—	Luo et al. (2021b)
*L.gatesiae*	LWZ20180515-3	MT319447	—	Wang et al. (2021)
*L.gatesiae*	LWZ20180515-32	MT319448	—	Wang et al. (2021)
*L.griseliniae*	KHL 12971 (GB)	DQ873651	—	Larsson et al. (2006)
*L.hengduanensis*	CLZhao 20627	OR793233	PP657611	Present study
*L.hengduanensis*	CLZhao 25551	OR658999	PP657610	Present study
*L.hengduanensis*	CLZhao 32713	OR899153	—	Present study
*L.hengduanensis*	CLZhao 32714	OR899154	—	Present study
*L.hengduanensis*	CLZhao 32782	OR899155	PP657612	Present study
*L.juniperi*	FR-0261086	KY081799	—	Riebesehl and Langer (2017)
*L.leptocystidiatus*	LWZ20170818-1	MT326514	—	Wang et al. (2021)
*L.leptocystidiatus*	LWZ20170818-2	MT326513	—	Wang et al. (2021)
*L.macrosporus*	CLZhao 4516	MN945977	—	Chen and Zhao (2020)
*L.mascarensis*	KAS-GEL4833	KY800399	—	Yurchenko et al. (2020)
*L.mascarensis*	KAS-GEL4908	KY800400	—	Yurchenko et al. (2020)
*L.microfasciculatus*	CLZhao 5109	MN954311	—	Chen and Zhao (2020)
*L.niveomarginatus*	CLZhao 16360	PP537949	PP657607	Present study
*L.niveus*	CLZhao 6431	MZ262541	MZ262526	Luo et al. (2021b)
*L.niveus*	CLZhao 6442	MZ262542	MZ262527	Luo et al. (2021b)
*L.ochraceoalbus*	CLZhao 4385	MZ262535	MZ262521	Luo et al. (2021b)
*L.ochraceoalbus*	CLZhao 4725	MZ262536	MZ262522	Luo et al. (2021b)
*L.ochraceoalbus*	MSK7247	KY800403	—	Yurchenko et al. (2017)
*L.orientalis*	GEL3376	DQ340325	—	Yurchenko et al. (2017)
*L.pruni*	GEL2327	DQ340312	—	Larsson et al. (2006)
*L.pruni*	Ryberg 021018 (GB)	DQ873624	—	Larsson et al. (2006)
*L.sambuci*	KAS-JR7	KY800402	KY795966	Yurchenko et al. (2017)
*L.sambuci*	83SAMHYP	JX857721	—	Yurchenko et al. (2017)
*L.vietnamensis*	TNM F9073	JX175044	—	Yurchenko et al. (2017)
*L.wuliangshanensis*	CLZhao 4108	MN945980	—	Chen and Zhao (2020)
*L.wuliangshanensis*	CLZhao 4167	MN945979	—	Chen and Zhao (2020)
*L.wumengshanensis*	CLZhao 29374	OR803021	PP657613	Present study
*L.wumengshanensis*	CLZhao 31486	OR899208	—	Present study
*L.wumengshanensis*	CLZhao 32705	OR899209	—	Present study
*L.wumengshanensis*	CLZhao 32736	OR899210	—	Present study
*L.wumengshanensis*	CLZhao 32800	OR899211	PP657614	Present study
*L.wumengshanensis*	CLZhao 32869	OR899212	—	Present study
*L.wumengshanensis*	CLZhao 32915	OR899213	PP657615	Present study
*L.yunnanensis*	CLZhao 2463	OP730711	OP730723	Guan et al. (2023)
*L.yunnanensis*	CLZhao 9375	OP730710	—	Guan et al. (2023)
*L.yunnanensis*	CLZhao 10041	OP730709	—	Guan et al. (2023)
*L.zhaotongensis*	CLZhao 32878	PP537950	PP657609	Present study
*Xylodonafromontanus*	H 7006811	OQ645463	—	Yurchenko et al. (2024)
*X.asiaticus*	CLZhao 10368	OM959479	—	Zhang et al. (2024)
*X.cystidiatus*	FR-0249200	MH880195	MH884896	Riebesehl et al. (2019)
*X.daweishanensis*	CLZhao 18492	OP730719	OP730727	Guan et al. (2023)
*X.daweishanensis*	CLZhao 18446	OP730717	OP730725	Guan et al. (2023)
*X.filicinus*	MSK-F 12869	MH880199	NG067836	Riebesehl et al. (2019)
*X.fissuratus*	CLZhao 7007	OP730713	—	Guan et al. (2023)
*X.fissuratus*	CLZhao 9407	OP730714	—	Guan et al. (2023)
*X.hastifer*	K(M) 172400	NR166558	—	Riebesehl and Langer (2017)
*X.hyphodontinus*	KAS-GEL9222	MH880205	MH884903	Riebesehl et al. (2019)
*X.macrosporus*	CLZhao 10226	MZ663809	MZ663817	Luo et al. (2021a)
*X.puerensis*	CLZhao 8142	OP730720	OP730728	Guan et al. (2023)
*X.puerensis*	CLZhao 8639	OP730721	OP730729	Guan et al. (2023)
*X.quercinus*	Larsson 11076 (GB)	KT361633	—	Larsson et al. (2004)
*X.ramicida*	Spirin 7664	NR138013	—	Unpublished
*X.subflaviporus*	Wu 0809-76	KX857803	—	Chen et al. (2017)
*X.tropicus*	CLZhao 3351	OL619261	OL619269	Qu et al. (2022)
*X.wenshanensis*	CLZhao 15729	OM338097	OM338104	Luo et al. (2022)
*X.xinpingensis*	CLZhao 11224	MW394662	MW394654	Luo et al. (2022)

**Figure 1.**
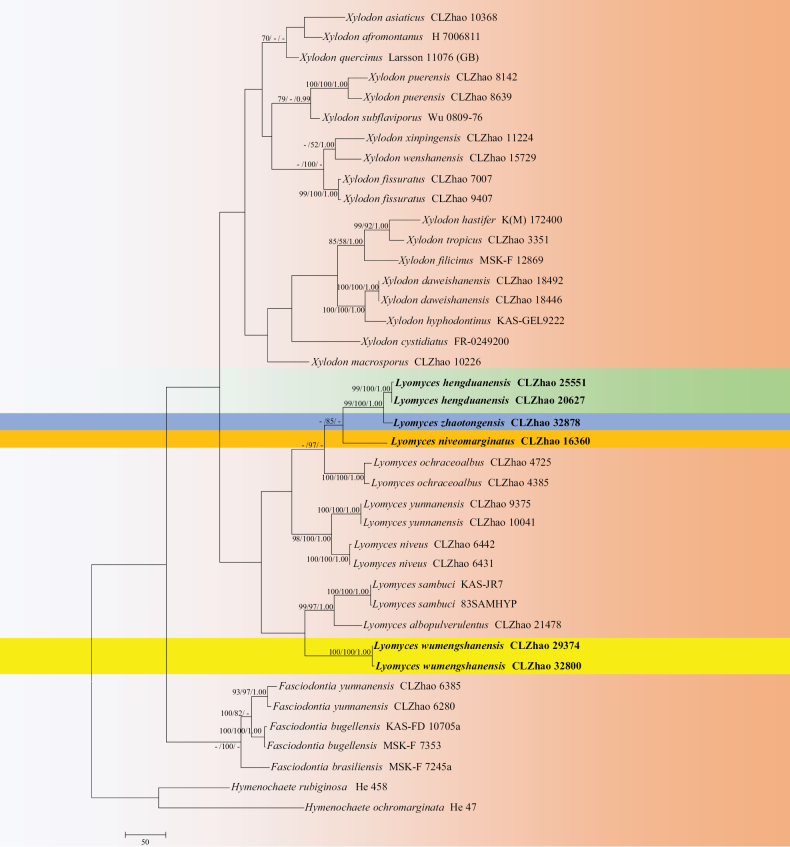
Maximum Parsimony strict consensus tree illustrating the phylogeny of four new species and related species in *Lyomyces* within Schizoporaceae, based on ITS+nLSU sequences. Branches are labelled with Maximum Likelihood bootstrap values ≥ 70%, parsimony bootstrap values ≥ 50% and Bayesian posterior probabilities ≥ 0.95, respectively.

**Figure 2.**
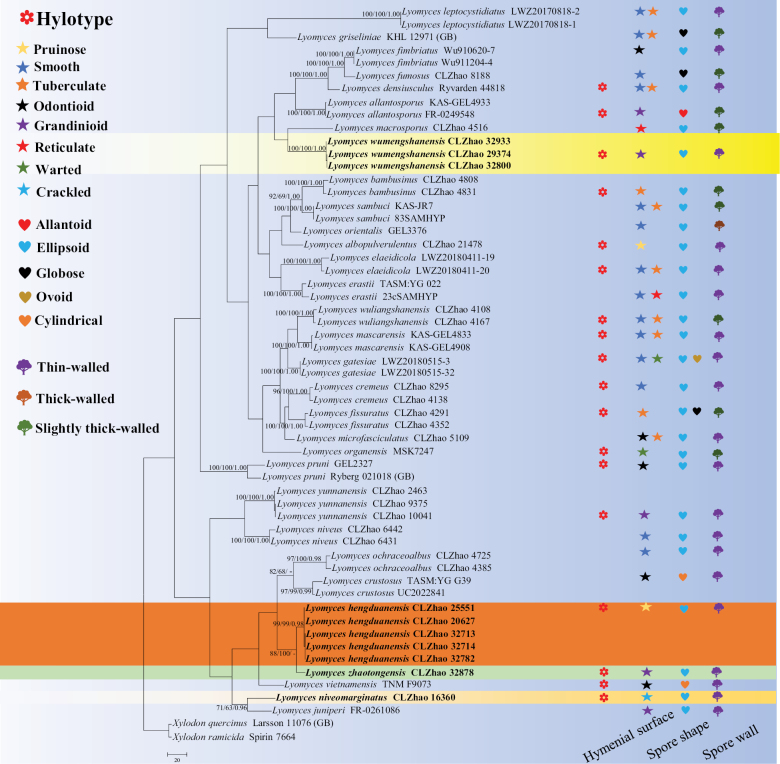
Maximum parsimony strict consensus tree illustrating the phylogeny of the four new species and related species in *Lyomyces*, based on ITS sequences. Branches are labelled with Maximum Likelihood bootstrap values > 70%, parsimony bootstrap values > 50% and Bayesian posterior probabilities > 0.95, respectively.

The sequences were aligned in MAFFT version 7 (Katoh et al. 2019) using the G-INS-i strategy. The alignment was adjusted manually using AliView version 1.27 (Larsson 2014). The sequence alignments were deposited in figshare (DOI: 10.6084/m9.figshare.27166521). Sequences of *Hymenochaeteochromarginata* P.H.B. Talbot and *Hymenochaeterubiginosa* (Dicks.) Lév., retrieved from GenBank, were used as the outgroups in the ITS+nLSU analysis (Fig. 1). The sequence alignments were deposited in figshare (DOI: 10.6084/m9.figshare.27166521). Sequences of *Xylodonquercinus* (Pers.) Gray and *Xylodonramicida* Spirin & Miettinen, retrieved from GenBank, were used as the outgroups in the ITS analysis (Fig. 2) (Guan et al. 2023; Zhang et al. 2024).

Maximum Parsimony (MP), Maximum Likelihood (ML) and Bayesian Inference (BI) analyses were applied to the combined three datasets following a previous study (Zhao and Wu 2017). All characters were equally weighted and gaps were treated as missing data. Trees were inferred using the heuristic search option with TBR branch swapping and 1,000 random sequence additions. Max-trees were set to 5,000, branches of zero length were collapsed and all parsimonious trees were saved. Clade robustness was assessed using bootstrap (BT) analysis with 1,000 pseudo-replicates (Felsenstein 1985). Descriptive tree statistics - tree length (TL), composite consistency index (CI), composite retention index (RI), composite rescaled consistency index (RC) and composite homoplasy index (HI) - were calculated for each maximum parsimonious tree generated. The combined dataset was also analysed using Maximum Likelihood (ML) in RAxML-HPC2 through the CIPRES Science Gateway (Miller et al. 2012). Branch support (BS) for the ML analysis was determined by 1000 bootstrap pseudo-replicates.

MrModelTest 2.3 (Nylander 2004) was used to determine the best-ﬁt evolution model for each dataset for Bayesian Inference (BI), which was performed using MrBayes 3.2.7a with a GTR+I+G model of DNA substitution and a gamma distribution rate variation across sites (Ronquist et al. 2012). A total of four Markov chains were run for two runs from random starting trees for 1.905 million generations for ITS+nLSU (Fig. 1) and 2 million generations for ITS (Fig. 2), with trees and parameters sampled every 1,000 generations. The ﬁrst quarter of all of the generations were discarded as burn-in. A majority rule consensus tree was computed from the remaining trees. Branches were considered as significantly supported if they received a Maximum Likelihood bootstrap support value (BS) of ≥ 70%, a Maximum Parsimony bootstrap support value (BT) of ≥ 70% or a Bayesian Posterior Probability (BPP) of ≥ 0.95.

## ﻿Results

### ﻿Molecular phylogeny

The ITS+nLSU dataset (Fig. 1) comprised sequences from 40 fungal specimens representing 29 taxa. The dataset had an aligned length of 2,112 characters, of which 1,298 characters were constant, 254 were variable and parsimony-uninformative and 560 were parsimony-informative. Maximum parsimony analysis yielded one equally parsimonious tree (TL = 2,513, CI = 0.4990, HI = 0.5010, RI = 0.6658 and RC = 0.3322). The best model of nucleotide evolution for the ITS+nLSU dataset estimated and applied in the Bayesian analysis was found to be GTR+I+G. Bayesian analysis and ML analysis resulted in a similar topology as in the MP analysis. The Bayesian analysis had an average standard deviation of split frequencies = 0.009992 (BI) and the effective sample size (ESS) across the two runs is double the average ESS (avg. ESS) = 2078.5. The phylogram, based on the ITS+nLSU rDNA gene regions (Fig. 1), included three genera within Schizoporaceae (Hymenochaetales), which were *Fasciodontia*, *Lyomyces* and *Xylodon*, in which four new species were grouped into the genera *Lyomyces*.

The ITS dataset (Fig. 2) comprised sequences from 57 fungal specimens representing 33 taxa. The dataset had an aligned length of 696 characters, of which 270 characters were constant, 41 were variable and parsimony-uninformative and 385 were parsimony-informative. Maximum parsimony analysis yielded 80 equally parsimonious tree (TL = 1,748, CI = 0.4027, HI = 0.5973, RI = 0.6935 and RC = 0.2793). The best model of nucleotide evolution for the ITS dataset estimated and applied in the Bayesian analysis was found to be GTR+I+G. Bayesian analysis and ML analysis resulted in a similar topology as in the MP analysis. The Bayesian analysis had an average standard deviation of split frequencies = 0.014964 (BI) and the effective sample size (ESS) across the two runs is double the average ESS (avg. ESS) = 1,387.5. The phylogenetic tree (Fig. 2), inferred from the ITS sequences, highlighted that *L.hengduanensis* group with *L.zhaotongensis*; and then closely grouped with *L.crustosus* (Pers.) P. Karst., *L.ochraceoalbus* C.L. Zhao and *L.vietnamensis* (Yurchenko & Sheng H. Wu) Riebesehl & Langer. *Lyomycesniveomarginatus* was retrieved as a sister to *L.juniperi* (Bourdot & Galzin) Riebesehl & Langer. *Lyomyceswumengshanensis* was retrieved as a sister to *L.macrosporus* C.L. Zhao. Moreover, *Lyomyceszhaotongensis* grouped with *L.hengduanensis* and closely clustered with *L.crustosus*, *L.ochraceoalbus* and *L.vietnamensis*.

### ﻿Taxonomy

#### ﻿ Lyomyces hengduanensis

Taxon classificationFungiPolyporalesCorticiaceae

Q. Yuan & C.L. Zhao sp. nov.

1E6DD629-D94E-5C23-8474-60E763399080

 853724

Figs 3 , 4

##### Type material.

***Holotype*.** China • Yunnan Province, Lincang, Fengqing County, Yaojie Town, GPS coordinates 24°66'N, 100°19'E, altitude 2060 m, on a fallen branch of angiosperm, leg. C.L. Zhao, 22 October 2022, CLZhao 25551 (SWFC).

**Figure 3.**
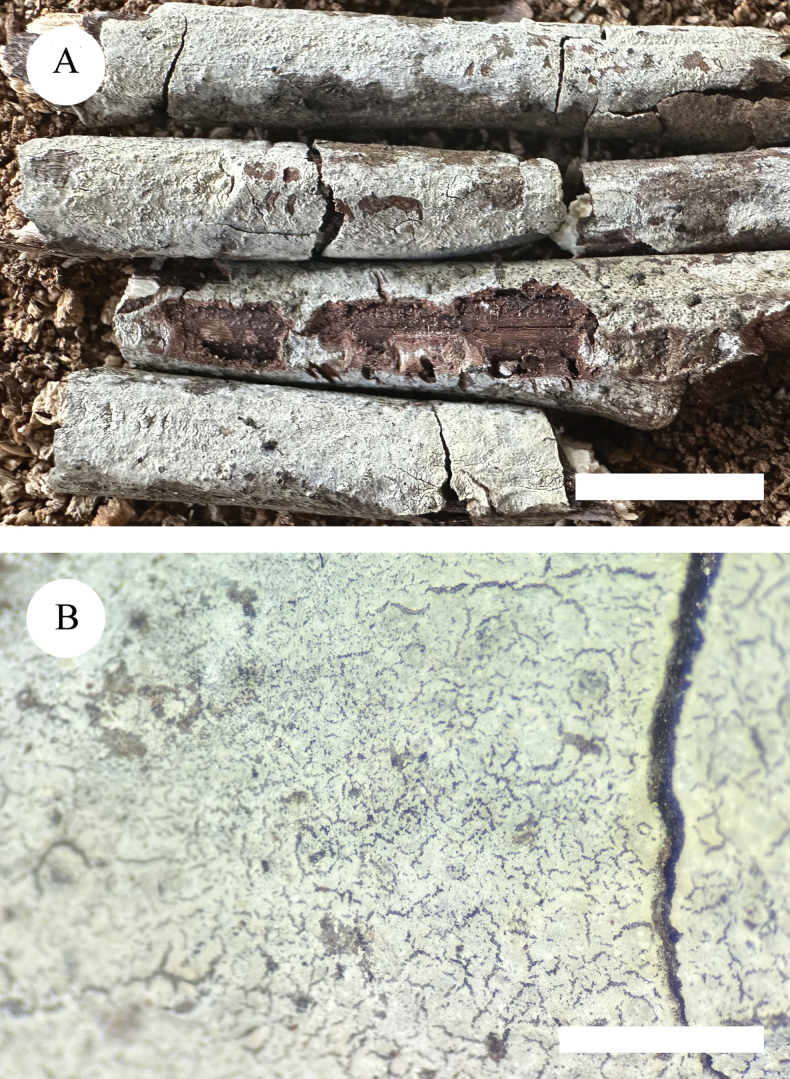
Basidiomata of *Lyomyceshengduanensis* (holotype). Scale bars: 1 cm (**A**); 2 mm (**B**).

##### Etymology.

*Hengduanensis* (Lat.) refers to the type locality “Hengduan Mountain”.

**Figure 4.**
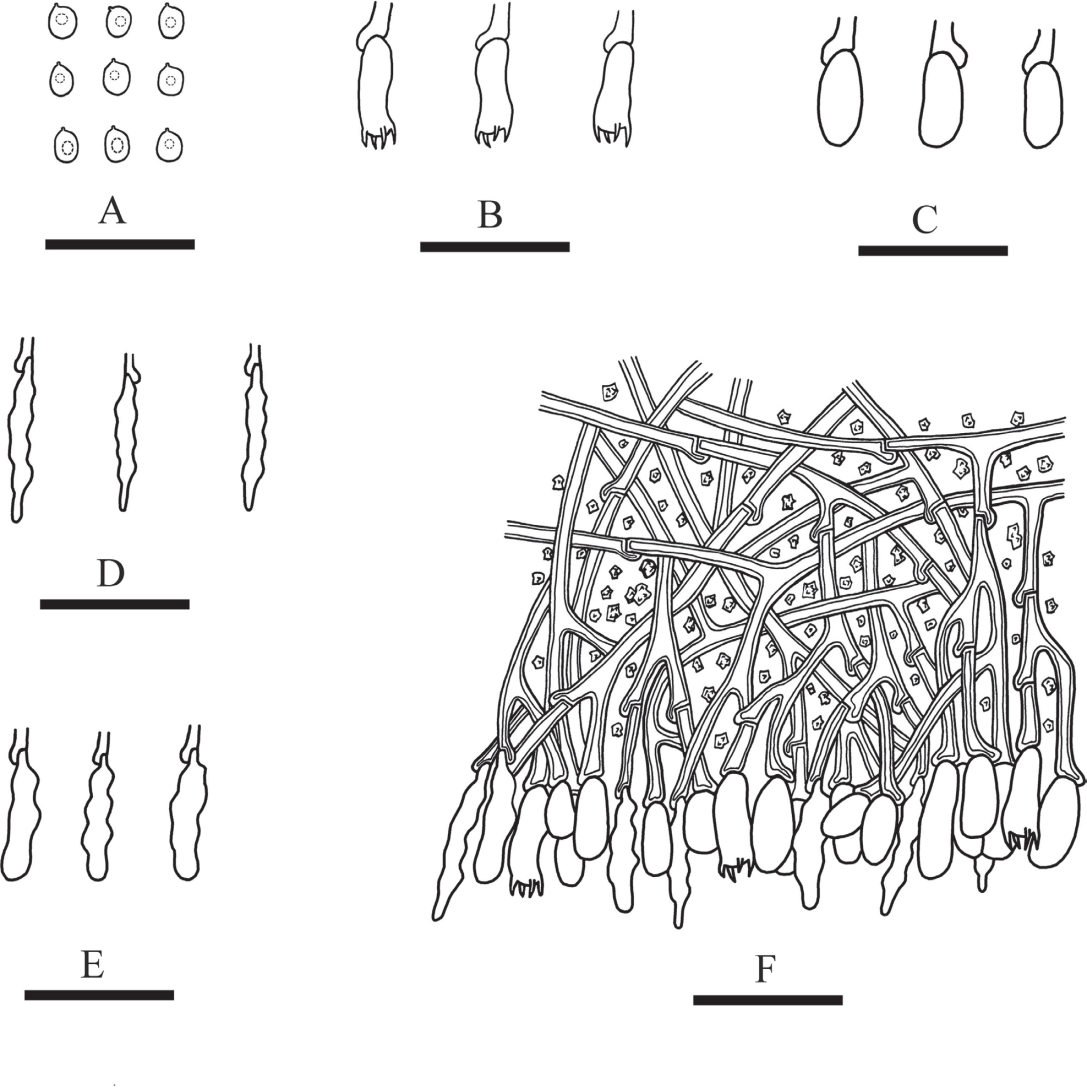
Microscopic structures of *Lyomyceshengduanensis* (holotype): basidiospores (**A**), basidia (**B**), basidioles (**C**), fusoid cystidia (**D**), subclavate cystidia (**E**), a section of hymenium (**F**). Scale bars: 20 µm (**A–F**).

##### Description.

Basidiomata annual, resupinate, adnate, brittle, without odour and taste when fresh and up to 3.5 cm long, 1 cm wide, 100 µm thick. Hymenial surface pruinose, white to cream when fresh, to cream to slightly buff upon drying. Sterile margin white to cream and up to 1 mm wide.

Hyphal system monomitic, generative hyphae with clamp connections, colourless, thick-walled, branched, 2–3 µm in diameter; IKI–, CB–, tissues unchanged in KOH. Numerous crystals present amongst generative hyphae.

Cystidia of two types: (1) fusoid, colourless, thin-walled, smooth, slightly constricted in the middle to somewhat sinuous, 17.5–25 × 3–4 µm; (2) subclavate, colourless, thin-walled, smooth, slightly constricted in the middle to somewhat sinuous, 16–23 × 3–4.5 µm; basidia clavate, with 4 sterigmata and a basal clamp connection, 10.5–14 × 3.5–5 µm.

Basidiospores ellipsoid, colourless, thin-walled, smooth, with one oil drop, CB–, IKI–, 3.5–6 × 3–4.5 µm, L = 4.63 µm, W = 3.65 µm, Q = 1.25–1.28 (n = 90/3).

##### Additional specimens examined

**(*paratypes*).** China • Yunnan Province, Zhaotong, Qiaojia County, Yaoshan Town, Yaoshan National Nature Reserve, 26°50'N, 102°59'E, altitude 2500 m, on a fallen branch of angiosperm, leg. C.L. Zhao, 22 August 2020, CLZhao 20627 (SWFC) • Zhaotong, Wumeng Mountain National Nature Reserve, GPS coordinates 27°72'N, 103°92'E, altitude 1424 m, on a fallen branch of angiosperm, leg. C.L. Zhao, 29 August 2023, CLZhao 32713, CLZhao 32714, CLZhao 32782 (SWFC).

#### ﻿ Lyomyces niveomarginatus

Taxon classificationFungiPolyporalesCorticiaceae

Q. Yuan & C.L. Zhao sp. nov.

11D7820E-D27B-52E5-A74E-E933F5F012F0

 853725

Figs 5 , 6

##### Type material.

***Holotype*.** China • Yunnan Province, Wenshan, Wenshan National Nature Reserve, GPS coordinates 23°21'N, 104°10'E, altitude 1950 m, on a fallen branch of angiosperm, leg. C.L. Zhao, 26 July 2019, CLZhao 16360 (SWFC).

**Figure 5.**
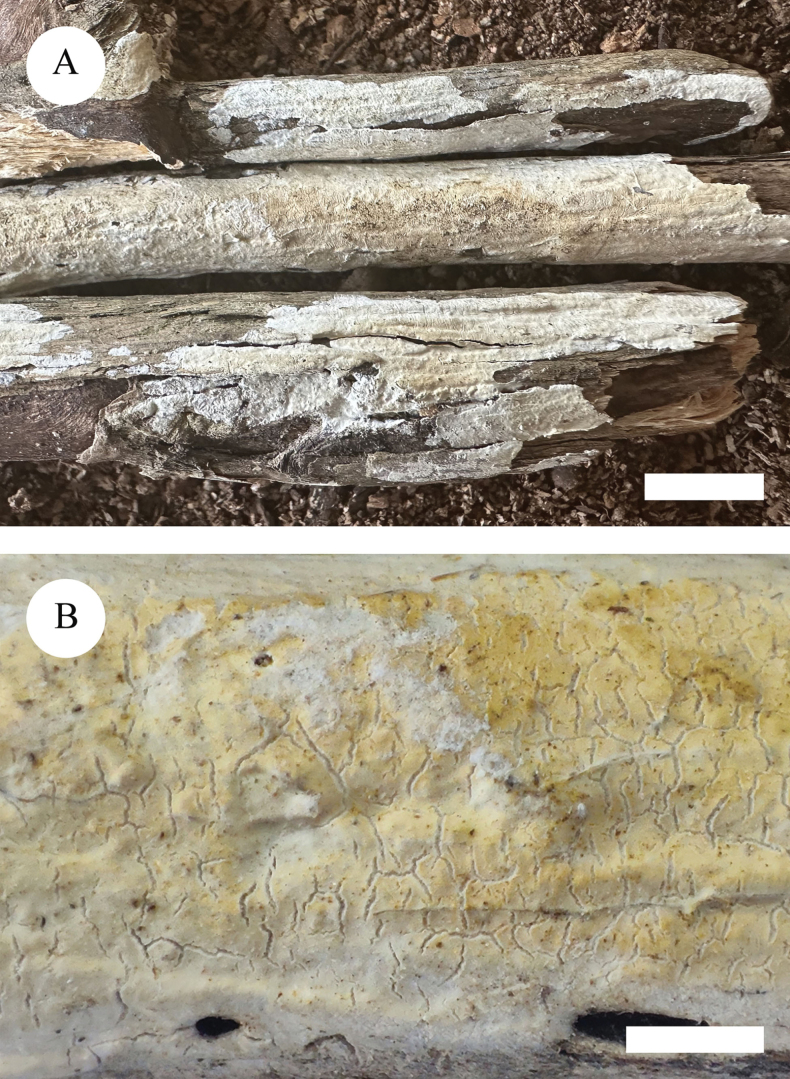
Basidiomata of *Lyomycesniveomarginatus* (holotype). Scale bars: 1 cm (**A**); 2 mm (**B**).

##### Etymology.

*Niveomarginatus* (Lat.) refers to the niveous margin of basidiomata.

**Figure 6.**
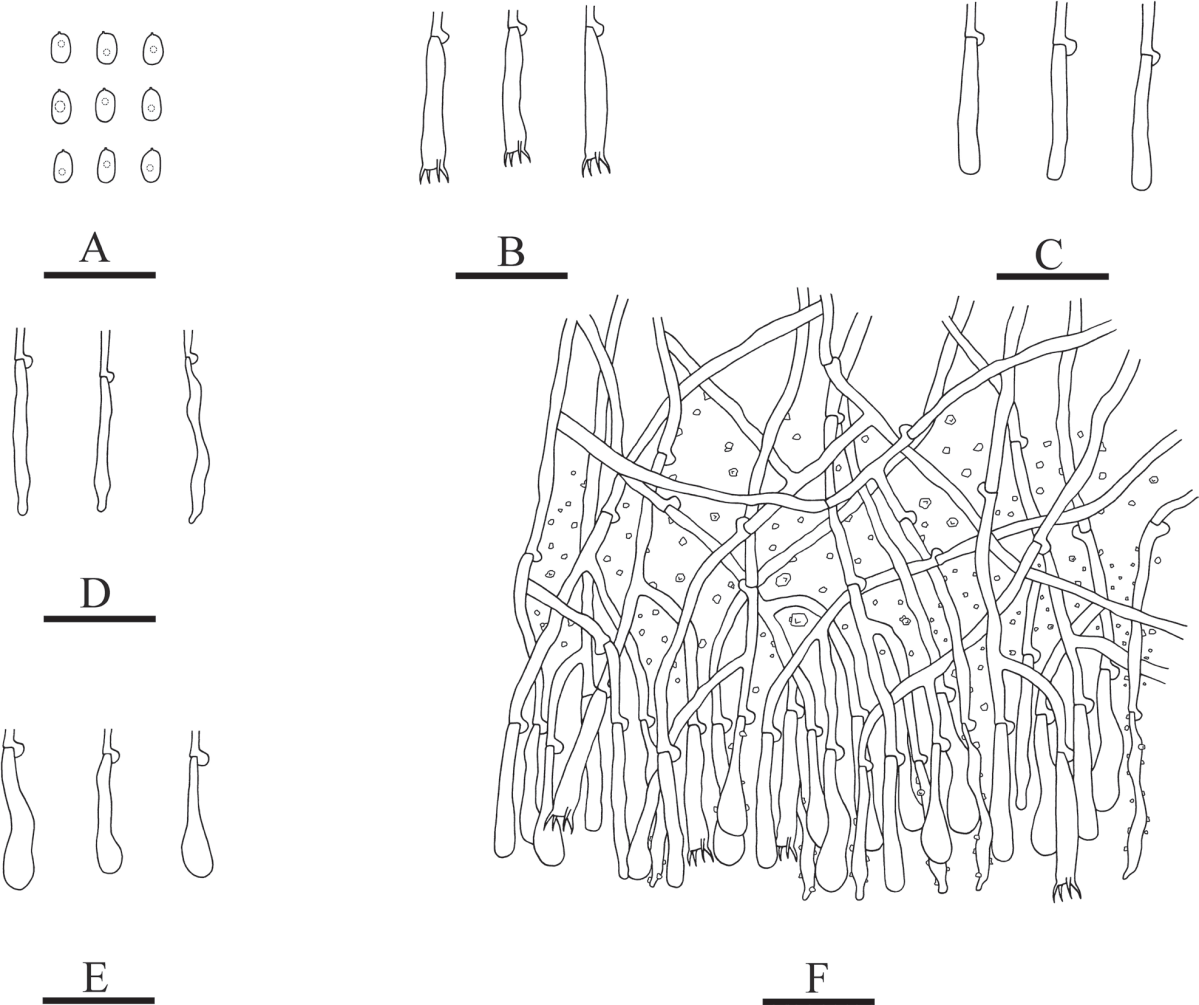
Microscopic structures of *Lyomycesniveomarginatus* (holotype): basidiospores (**A**), basidia (**B**), basidioles (**C**), fusoid cystidia (**D**), clavate cystidia (**E**), a section of hymenium (**F**). Scale bars: 20 µm (**A–F**).

##### Description.

Basidiomata annual, resupinate, adnate, subceraceous, without odour and taste when fresh and up to 7.5 cm long, 2 cm wide, 150 µm thick. Hymenial surface crackled, white to cream when fresh, to cream to slightly buff upon drying. Sterile margin distinct, whitish and up to 2 mm wide.

Hyphal system monomitic, generative hyphae with clamp connections, colourless, thin-walled, branched, 1.5–3.5 µm in diameter; IKI–, CB–, tissues unchanged in KOH. Numerous crystals present amongst generative hyphae.

Cystidia of two types: (1) fusoid, colourless, thin-walled, smooth, 25–29 × 2–3 µm; (2) clavate, colourless, thin-walled, smooth, 20–25.5 × 4.5–5.5 µm; basidia subclavate, with 4 sterigmata and a basal clamp connection, 23–29 × 2.5–3.5 µm.

Basidiospores ellipsoid, colourless, thin-walled, smooth, with one oil drop, CB–, IKI–, 4.5–7 × (2.5–)3–4 µm, L = 5.51 µm, W = 3.15 µm, Q = 1.75 (n = 30/1).

##### Additional specimens examined

**(*paratypes*).** China • Yunnan Province, Wenshan, Wenshan National Nature Reserve, GPS coordinates 23°21'N, 104°10'E, altitude 1950 m, on a fallen branch of angiosperm, leg. C.L. Zhao, 7 August 2024, CLZhao 40333, CLZhao 40334 (SWFC).

#### ﻿ Lyomyces wumengshanensis

Taxon classificationFungiPolyporalesCorticiaceae

Q. Yuan & C.L. Zhao sp. nov.

C1A8972E-0E45-528C-9DE2-3F8D00F4F9C0

 853726

Figs 7 , 8

##### Type material.

***Holotype*.** China • Yunnan Province, Zhaotong, Daguan County, Wumeng Mountain National Nature Reserve, GPS coordinates 27°72'N, 103°92'E, altitude 1424 m, on a fallen branch of angiosperm, leg. C.L. Zhao, 3 July 2023, CLZhao 29374 (SWFC).

**Figure 7.**
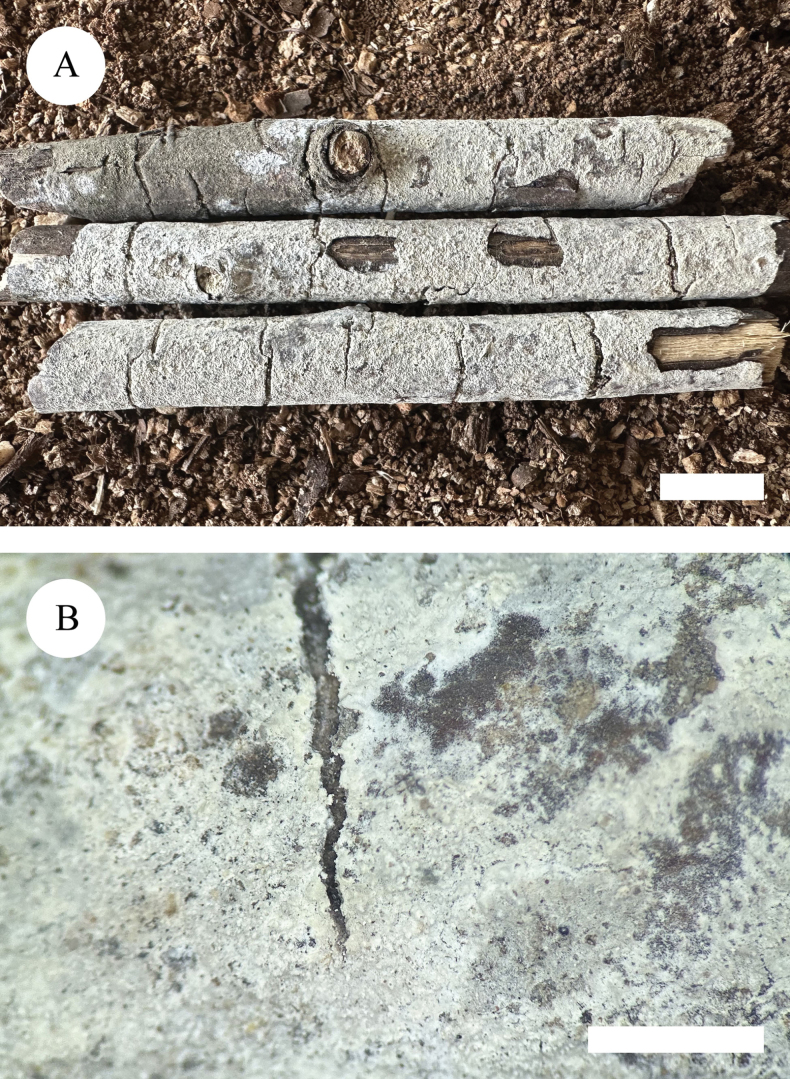
Basidiomata of *Lyomyceswumengshanensis* (holotype). Scale bars: 1 cm (**A**); 2 mm (**B**).

##### Etymology.

*Wumengshanensis* (Lat.) refers to the type locality “Wumeng Mountain”.

**Figure 8.**
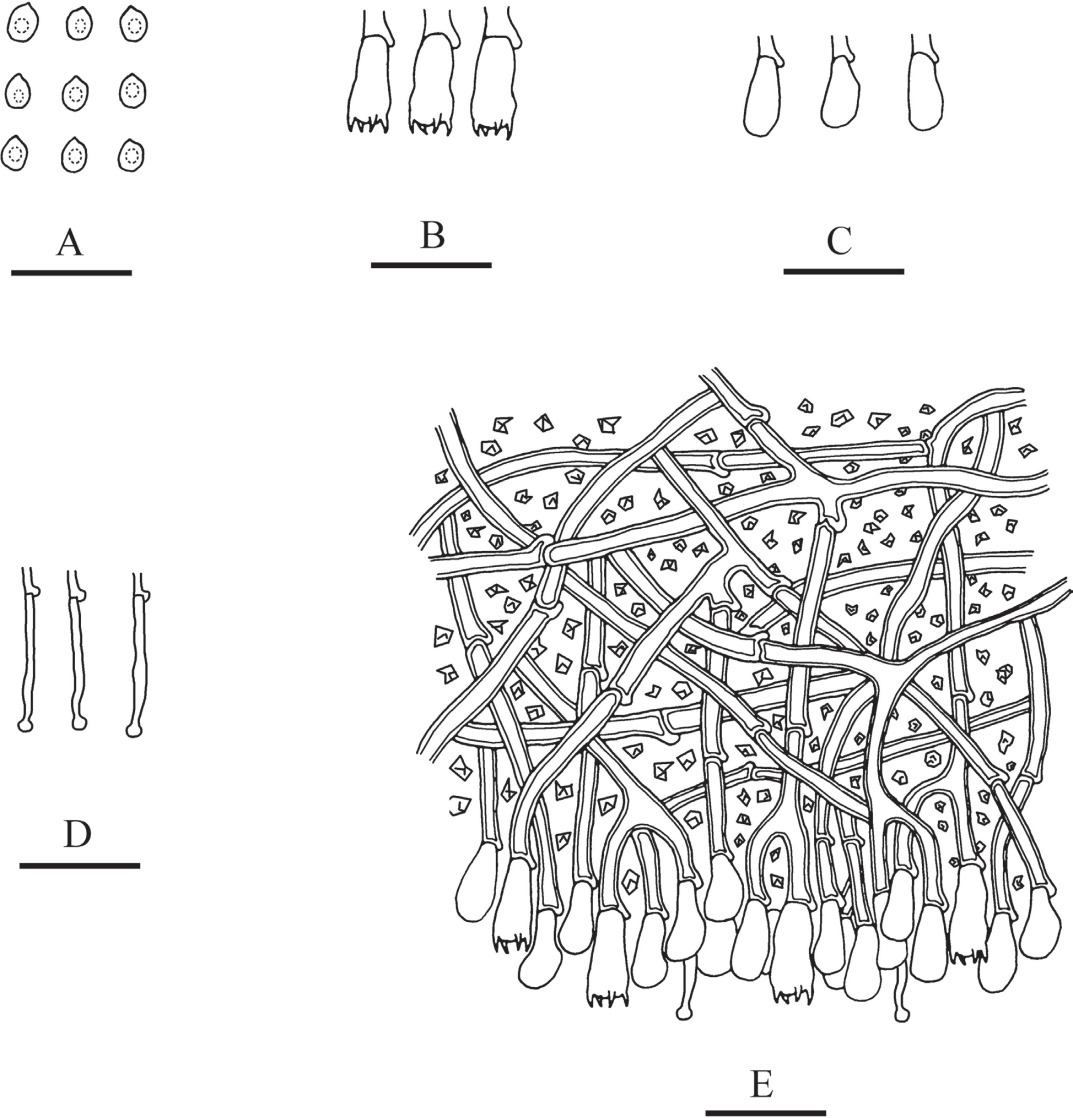
Microscopic structures of *Lyomyceswumengshanensis* (holotype): basidiospores (**A**), basidia (**B**), basidioles (**C**), capitate cystidia (**D**), a section of hymenium (**E**). Scale bars: 10 µm (**A–E**).

##### Description.

Basidiomata annual, resupinate, adnate, coriaceous when fresh, becoming farinaceous upon drying, without odour and taste when fresh and up to 5 cm long, 2 cm wide, 150 µm thick. Hymenial surface grandinioid, white when fresh, to cream upon drying. Sterile margin white and up to 1 mm wide.

Hyphal system monomitic, generative hyphae with clamp connections, colourless, thick-walled, branched, 3–4 µm in diameter; IKI–, CB–, tissues unchanged in KOH. Numerous crystals present amongst generative hyphae.

Cystidia capitate, colourless, thin-walled, smooth, 24.5–29 × 3–4 µm; basidia subclavate to barrelled, colourless, with 4 sterigmata and a basal clamp connection, 11.5–14 × 5.5–6.5 µm.

Basidiospores ellipsoid to broad ellipsoid, colourless, thin-walled, smooth, with one oil drop, CB–, IKI–, 4–6 × 3–5 µm, L = 5.4 µm, W = 4.2 µm, Q = 1.28–1.32 (n = 120/4).

##### Additional specimen examined

**(*paratype*).** China • Yunnan Province, Zhaotong, Wumeng Mountain National Nature Reserve, 27°72'N, 103°92'E, altitude 1424 m, on a fallen branch of angiosperm, leg. C.L. Zhao, 29 August 2023, CLZhao 31486, CLZhao 32705, CLZhao 32736, CLZhao 32800, CLZhao 32869, CLZhao 32915, CLZhao 32933 (SWFC).

#### ﻿ Lyomyces zhaotongensis

Taxon classificationFungiPolyporalesCorticiaceae

Q. Yuan & C.L. Zhao sp. nov.

9ABE5EE2-E572-5478-BC8F-00CBB8C47184

 853727

Figs 9 , 10

##### Type material.

***Holotype*.** China •Yunnan Province, Zhaotong, Wumeng Mountain National Nature Reserve, GPS coordinates 27°77'N, 104°29'E, altitude 2900 m, on the fallen branch of angiosperm, leg. C.L. Zhao, 29 August 2023, CLZhao 32878 (SWFC).

**Figure 9.**
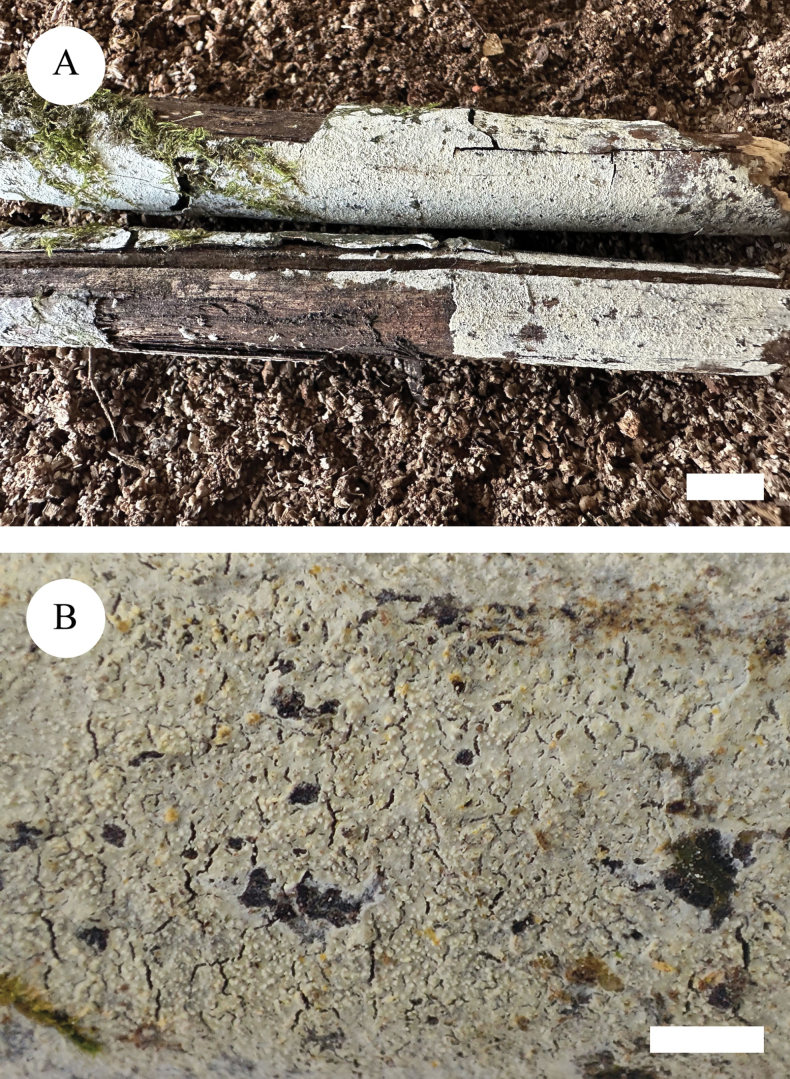
Basidiomata of *Lyomyceszhaotongensis* (holotype). Scale bars: (**A**) 1 cm; (**B**) 2 mm.

##### Etymology.

*Zhaotongensis* (Lat.) refers to the type locality “Zhaotong”.

**Figure 10.**
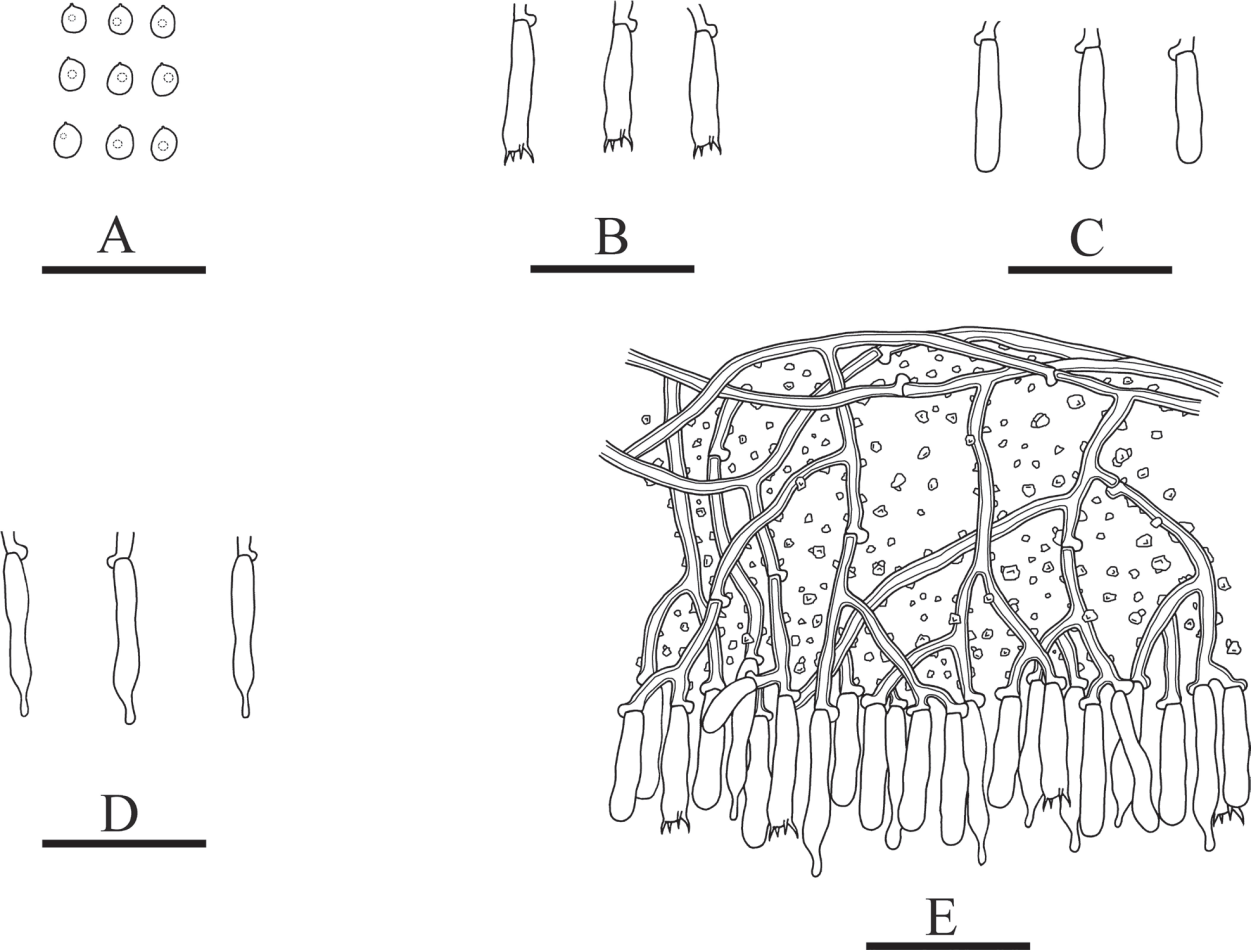
Microscopic structures of *Lyomyceszhaotongensis* (holotype): basidiospores (**A**), basidia (**B**), basidioles (**C**), fusoid cystidia (**D**), a section of hymenium (**E**). Scale bars: 20 µm (**A–E**).

##### Description.

Basidiomata annual, resupinate, adnate, farinaceous when fresh, becoming coriaceous upon drying and up to 9.5 cm long, 3 cm wide, 30–80 um thick. Hymenial surface grandinioid, cream when fresh and cream to buff upon drying. Sterile margin white to cream and up to 1 mm wide.

Hyphal system monomitic, generative hyphae with clamp connections, colourless, thick-walled, branched, 1.5–2 µm in diameter; IKI–, CB–, tissues unchanged in KOH. Numerous crystals present amongst generative hyphae.

Cystidia fusoid, colourless, thin-walled, smooth, 16–20.5 × 2.5–3.5 µm. Basidia clavate, with 4 sterigmata and a basal clamp connection, 14–16.5 × 2.5–3.5 µm.

Basidiospores broadly ellipsoid, colourless, thin-walled, smooth, with oil drops, CB–, IKI–, 2.6–3.5 × 2.5–3 µm, L = 2.99 µm, W = 2.75 µm, Q = 1.08 (n = 30/1).

##### Additional specimen examined

**(*paratype*).** China • Yunnan Province, Zhaotong, Wumeng Mountain National Nature Reserve, GPS coordinates 27°77'N, 104°29'E, altitude 2900 m, on the fallen branch of angiosperm, leg. C.L. Zhao, 10 August 2024, CLZhao 40335 (SWFC).

## ﻿Discussion

Many recently new wood-inhabiting fungal taxa have been reported in the subtropics and tropics, including in the genus *Lyomyces* (Xiong et al. 2009; Chen et al. 2017; Kan et al. 2017a, b; Riebesehl and Langer 2017; Viner et al. 2018; Chen and Zhao 2020; Luo et al. 2021a, b, c, 2022; Qu and Zhao 2022; Qu et al. 2022; Viner et al. 2022; Guan et al. 2023; Deng et al. 2024a, b; Zhang et al. 2024). Prior to this study, the following sixteen *Lyomyces* species were reported from China as *L.albopulverulentus* C.L. Zhao, *L.albus* (Sheng H. Wu) Riebesehl & Langer, *L.bambusinus*, *L.capitatocystidiatus* (H.X. Xiong, Y.C. Dai & Sheng H. Wu) Riebesehl & Langer, *L.cremeus* C.L. Zhao, *L.fissuratus*, *L.fumosus*, *L.leptocystidiatus* Xue W. Wang & L.W. Zhou, *L.macrosporus* C.L. Zhao & K.Y. Luo, *L.microfasciculatus* (Yurchenko & Sheng H. Wu) Riebesehl & Langer, *L.niveus*, *L.ochraceoalbus*, *L.sambuci*, *L.tenuissimus* (Yurchenko & Sheng H. Wu) Riebesehl & Langer, *L.wuliangshanensis* C.L. Zhao and *L.yunnanensis* C.L. Zhao (Xiong et al. 2009; Yurchenko et al. 2013; Riebesehl and Langer 2017; Chen and Zhao 2020; Luo et al. 2021b, c; Wang et al. 2021). The present study reports four new species in the genus *Lyomyces*, based on a combination of morphological features and molecular evidence.

Phylogenetically, based on the multiple loci in *Hyphodontia* s.l., six genera of *Fasciodontia*, *Hastodontia*, *Hyphodontia*, *Lyomyces*, *Kneiffiella* and *Xylodon*, were divided into four clades in the wood-inhabiting fungal order Hymenochaetales (Wang et al. 2021). In the present study, the phylogram inferred from the ITS+nLSU data, four new species grouped into the genus *Lyomyces* (Fig. 1). Based on ITS topology (Fig. 2), in which *L.hengduanensis* group with *L.zhaotongensis* and then closely grouped with *L.crustosus*, *L.ochraceoalbus* and *L.vietnamensis*. *Lyomycesniveomarginatus* was retrieved as a sister to *L.juniperi*. *L.wumengshanensis* was sister to *L.macrosporus*. Moreover, *L.zhaotongensis* grouped with *L.hengduanensis* and then closely clustered with three species: *L.crustosus*, *L.ochraceoalbus* and *L.vietnamensis*. However, morphologically, *L.zhaotongensis* can be delimited from *L.hengduanensis* by its the grandinioid hymenial surface and longer basidia (14–16.5 × 2.5–3.5 µm); *L.crustosus* can be separated from *L.hengduanensis* by its odontioid hymenial surface and narrow basidiospores (5–7.5 × 2.5–3 µm) (Lentz and McKay 1976); *L.ochraceoalbus* differs in *L.hengduanensis* by having a smooth hymenial surface and lacking a cystidium (Luo et al. 2021c); *L.vietnamensis* differs from *L.hengduanensis* by its aculeate hymenial surface and narrow basidiospores (5.8–6.1 × 2.6–2.9 µm; Yurchenko and Wu (2013)). *L.juniperi* can be delimited from *L.niveomarginatus* by its smooth hymenial surface with some scattered small granules and wider basidia (15–25 × 4–4.5 µm; Hjortstam and Ryvarden (2004)); *L.macrosporus* can be separated from *L.wumengshanensis* by its reticulate hymenial surface and longer basidiospores (6.7–8.9 × 4.4–5.4 µm; Chen and Zhao (2020)); *L.crustosus* can be delimited from *L.zhaotongensis* by its odontioid hymenial surface and longer basidiospores (5–7.5 × 2.5–3 µm; Lentz and McKay (1976)); *L.hengduanensis* can be delimited from *L.zhaotongensis* by its pruinose hymenial surface and shorter basidia (14–16.5 × 2.5–3.5 µm); *L.ochraceoalbus* differs in *L.zhaotongensis* by having smooth hymenial surface and longer basidiospores (4–5 × 2.5–3.5 µm; Luo et al. (2021c)); *L.vietnamensis* can be delimited from *L.zhaotongensis* by its aculeate hymenial surface and longer basidiospores (5.8–6.1 × 2.6–2.9 µm; Yurchenko and Wu (2013)).

Morphologically, *Lyomyceshengduanensis* resembles four taxa viz. *L.albopulverulentus*, *L.bambusinus*, *L.mascarensis* Riebesehl, Yurch. & Langer and *L.yunnanensis*, by the similar ellipsoid basidiospores. However, *L.albopulverulentus* differs from *L.hengduanensis* by its larger basidia (24.5–28.5 × 7–9 µm) and basidiospores (8–10.5 × 5.5–7 µm; Guan et al. (2023)); *L.bambusinus* can be separated from *L.hengduanensis* by its colliculose to tuberculate hymenial surface and longer basidia (16.5–35 × 3.5–7 µm; Chen and Zhao (2020)); *L.mascarensis* is distinct from *L.hengduanensis* by having indistinctly colliculose hymenial surface and longer basidia (16–17.5 × 3.5–4.5 µm; Yurchenko et al. (2017)); *L.yunnanensis* is distinguished from *L.hengduanensis* by its grandinioid hymenial surface and longer basidia (16.5–27 × 4–5.5 µm; Guan et al. (2023)).

Morphologically, *Lyomycesniveomarginatus* resembles several species viz. *L.albopulverulentus*, *L.cremeus*, *L.macrosporus*, *L.wuliangshanensis* and *L.yunnanensis* by the cream to buff hymenial surface and ellipsoid basidiospores. However, *L.albopulverulentus* differs from *L.niveomarginatus* by its pruinose hymenial surface and wider basidia (24.5–28.5 × 7–9 µm; Guan et al. (2023)); *L.cremeus* can be separated from *L.niveomarginatus* by its smooth hymenial surface and shorter basidia (9–18.5 × 3–6 µm; Chen and Zhao (2020)); *L.macrosporus* differs from *L.niveomarginatus* by its reticulate hymenial surface and wider basidia (23–29 × 2.5–3.5 µm) and wider basidiospores (6.7–8.9 × 4.4–5.4 µm; Chen and Zhao (2020)); *L.wuliangshanensis* can be delimited from *L.niveomarginatus* by its smooth to more or less tuberculate hymenial surface and shorter basidia (12–20 × 3–4.3 µm; Chen and Zhao (2020)); *L.yunnanensis* is distinct from *L.niveomarginatus* by having grandinioid hymenial surface and wider basidia (16.5–27 × 4–5.5 µm; Guan et al. (2023)).

Morphologically, *Lyomyceswumengshanensis* resembles *L.bambusinus*, *L.cremeus*, *L.fumosus*, *L.fissuratus*, *L.wuliangshanensis* and *L.yunnanensis* by having the capitate cystidia. However, *L.bambusinus* is distinct from *L.wumengshanensis* by possessing tapering cystidia (40–65 × 4–5.5 µm) and longer basidia (16.5–35 × 3.5–7 µm; Chen and Zhao (2020)); *L.cremeus* differs from *L.wumengshanensis* by its smooth hymenial surface and possesses tapering cystidia (18–35 × 3–4.5 µm; Chen and Zhao (2020)); *L.fumosus* can be separated from *L.wumengshanensis* by its smooth, smoky grey hymenial surface and narrower basidia (11.5–17.5 × 3–5 µm; Luo et al. (2021b)); *L.fissuratus* can be delimited from *L.wumengshanensis* by its longer and narrower basidia (14.7–23.3 × 2.9–4.8 µm; Luo et al. (2021b)); *L.wuliangshanensis* differs from *L.wumengshanensis* by its smooth to more or less tuberculate hymenial surface and narrower basidia (12–20 × 3–4.3 µm; Chen and Zhao (2020)); *L.yunnanensis* is separated from *L.wumengshanensis* by the longer basidia (16.5–27 × 4–5.5 µm) and possessing fusiform cystidia (18–39 × 4–6 µm; Guan et al. (2023)).

Morphologically, *Lyomyceszhaotongensis* reminds *L.albopulverulentus*, *L.cremeus*, *L.denudatus* Viner, *L.macrosporus* and *L.wuliangshanensis* by having the ellipsoid basidiospores. However, *L.albopulverulentus* can be separated from *L.zhaotongensis* by its pruinose hymenial surface and larger basidia (24.5–28.5 × 7–9 µm) and larger basidiospores (8–10.5 × 5.5–7 µm; Guan et al. (2023)); *L.cremeus* is distinct from *L.zhaotongensis* by its smooth hymenial surface and larger basidiospores (4.5–5.6 × 3.3–4.3 µm; Chen and Zhao (2020)); *L.denudatus* is separated from *L.zhaotongensis* by the smooth hymenial surface and longer basidiospores (4.8–7 × 2.8–4.2 µm; Viner and Miettinen (2022)); *L.macrosporus* differs from *L.zhaotongensis* due to its reticulate hymenial surface and larger basidia (22.2–38 × 4.5–7 µm) and larger basidiospores (6.7–8.9 × 4.4–5.4 µm; Chen and Zhao (2020)); *L.wuliangshanensis* can be delimited from *L.zhaotongensis* by its smooth to more or less tuberculate hymenial surface and longer basidiospores (3.5–5.3 × 2.8–4 µm; Chen and Zhao (2020)). A morphological comparison amongst four new *Lyomyces* species and seven similar species are presented in Table 2.

**Table 2.** A morphological comparison between four new *Lyomyces* species and seven similar species in the genus *Lyomyces*. The bold are new taxa.

Species name	Hymenial surface	Generative hyphae	Cystidia	Basidia	Basidiospores	References
*Lyomycesalbopulverulentus*	Pruinose/ white	Thick-walled/frequently branched	Capitate, 37–54 × 5–9 µm	Clavate, 24.5–28.5 × 7–9 µm	Ellipsoid, (7.5–)8–10.5(–11) × (5–)5.5–7 µm	Guan et al. (2023)
*Lyomycesbambusinus*	Colliculose to tuberculate/ cream to buff	Thick-walled/ branched	Capitate, 35–55 × 4–7 µm; tapering, 40–65 × 4–5.5 µm, cystidioles, 12–17 × 2–3 µm	Clavate, 16.5–35 × 3.5–7 µm	Broadly ellipsoid, (4.5–)4.7–5.9 (–6.2) × (3.4–)3.7–4.6(–4.8) µm	Chen and Zhao (2020)
*Lyomycescremeus*	Smooth/ pale cream	Thick-walled/ branched	Capitate, 20–40 × 3–5 µm; tapering, 18–35 × 3–4.5 µm	Clavate, 9–18.5 × 3–6 µm	Ellipsoid, 4.5–5.6(–5.8) × 3.3–4.3(–4.5) µm	Chen and Zhao (2020)
*Lyomycesdenudatus*	Smooth/ cream	Thin-walled to slightly thick-walled	Capitate, (21–)34.9–62 × (3.5–)4–5.5(–7) μm	Suburniform, 15–21.1(–25) × 3.8–5.5 μm	Ellipsoid, (4.1–)4.8–7 × 2.8–4.2(–4.7) μm	Viner and Miettinen (2022)
***Lyomyceshengduanensis***	Pruinose/ cream to slightly buff	Thick-walled/ branched	Fusoid, 17.5–25 × 3–4 µm; subclavate, 16–23 × 3–4.5 µm	Clavate, 10.5–14 × 3.5–5 µm	Ellipsoid, 3.5–6 × 3–4.5 µm	**Present study**
*Lyomycesmascarensis*	Smooth / cream or brownish	Thin-walled	Capitate, 17–38 × 3.5–6(–7) µm; submoniliform, 18–22 × 5–5.5 µm; tapering, 25–30 × 3.5–4.5 µm	Subcylindrical with one constriction, 16–17.5(–19) × 3.5–4.5(–6) µm	Ellipsoid or broadly ellipsoid, (4–)4.5–6 × (3–)3.3–4 µm	Yurchenko et al. (2017)
***Lyomycesniveomarginatus***	Smooth / cream to slightly buff	Thin-walled, branched	Fusoid, 25–29 × 2–3 µm; clavate, 20–25.5 × 4.5–5.5 µm	Subclavate, 23–29 × 2.5–3.5 µm	Ellipsoid, 4.5–7 × (2.5–)3–4 µm	**Present study**
*Lyomyceswuliangshanensis*	Tuberculate/ cream to buff	Thick-walled/ branched	Capitate, 22–37 × 3–6 µm; tapering, 21–35 × 4–6.5 µm	Clavate, 12–20 × 3–4.3 µm	Ellipsoid, (3.3–)3.5–5.3(–5.5) × 2.8–4(–4.2) µm	Chen and Zhao (2020)
***Lyomyceswumengshanensis***	Grandinioid/ white to cream	Thick-walled/ branched	Capitate, 24.5–29 × 3–4 µm	Subclavate to barreled, 11.5–14 × 5.5–6.5 µm	Ellipsoid to broad ellipsoid, 4–6 × 3–5 µm	**Present study**
*Lyomycesyunnanensis*	Grandinioid/ cream to buff	Thick-walled, frequently branched	Tapering, 18–39 × 4–6 µm; capitate, 16–23.5 × 3–5 µm	Clavate, 16.5–27 × 4–5.5 µm	Ellipsoid, (4.5–)5–7 × 3–4.5 µm	Guan et al. (2023)
***Lyomyceszhaotongensis***	Grandinioid/ cream to buff	Thick-walled/ branched	Fusoid, 16–20.5 × 2.5–3.5 µm	Clavate, 14–16.5 × 2.5–3.5 µm	Broadly ellipsoid, 2.6–3.5 × 2.5–3 µm	**Present study**

The Basidiomycota is a major phylum of the kingdom Fungi (He et al. 2019; Wijayawardene et al. 2020; Yuan et al. 2023; He et al. 2024), in which the wood-inhabiting fungi are an extensively studied group of Basidiomycota (Gilbertson and Ryvarden 1987; Bernicchia and Gorjón 2010; Núñez and Ryvarden 2001; Dai 2012; Ryvarden and Melo 2014; Wu et al. 2022b; Zhao et al. 2023; Dong et al. 2024), but the wood-inhabiting fungal diversity is still not well known in China, especially in subtropical and tropical areas, and many recently-described taxa of this ecologically important group were from China (Zhao et al. 2014; Zhao et al. 2015; Zhao et al. 2016; Bian et al. 2016; Ma and Zhao 2019; Guan et al. 2020; Huang and Zhao 2020; Guan et al. 2023; Ji et al. 2023; Liu et al. 2023; Yang et al. 2023; Deng et al. 2024a, b; Yang et al. 2024; Zhang et al. 2024; Zhou et al. 2024). Four new species in the present study are described, based on morphological and molecular phylogenetic analyses, also from the subtropics. This study enriches the wood-inhabiting fungal diversity in China and the world.

## Supplementary Material

XML Treatment for Lyomyces hengduanensis

XML Treatment for Lyomyces niveomarginatus

XML Treatment for Lyomyces wumengshanensis

XML Treatment for Lyomyces zhaotongensis

## ﻿Additional information

### Author ORCIDs

Qi Yuan https://orcid.org/0000-0002-6732-1656

Yunchao Li https://orcid.org/0009-0007-3500-3761

Yunfei Dai https://orcid.org/0009-0007-7734-4142

Kunyan Wang https://orcid.org/0009-0004-1678-9963

Changlin Zhao https://orcid.org/0000-0002-8668-1075

### Data availability

All of the data that support the findings of this study are available in the main text.
